# Cdk1-dependent phosphorylation of KIF4A at S1186 triggers lateral chromosome compaction during early mitosis

**DOI:** 10.1371/journal.pone.0209614

**Published:** 2018-12-21

**Authors:** Hideaki Takata, Marliza Madung, Kaoru Katoh, Kiichi Fukui

**Affiliations:** 1 Biomedical Research Institute, National Institute of Advanced Industrial Science and Technology (AIST), Ikeda, Osaka, Japan; 2 Department of Biotechnology, Graduate School of Engineering, Osaka University, Suita, Osaka, Japan; Institut de Genetique et Developpement de Rennes, FRANCE

## Abstract

Chromosome organization during cell division is achieved through the timely association of proteins with chromatin and is regulated by protein phosphorylation. Kinesin family member 4A (KIF4A) plays an important role in the chromosome organization through the formation of the chromosome scaffold structure. However, the relationship between the function of KIF4A and its phosphorylation remains unclear. Here, we demonstrate that Cdk1-dependent phosphorylation of KIF4A at S1186 is required for chromosome binding and chromosome scaffold formation. The KIF4A mutant, which is not phosphorylated at S1186, was found to localize to the nucleus during interphase but did not accumulate in the chromosome scaffold after nuclear envelope breakdown. In addition, defects in KIF4A phosphorylation were found to disrupt the interaction of KIF4A with the condensin I complex. As a result, the morphology of the chromosomes was observed to be laterally decondensed, without condensin I in the chromosome scaffold. Additionally, a defect in chromosome segregation, chromosome bridge formation, was often observed. Although both KIF4A and condensin I disappeared from the chromosomes, the chromosomal localization of condensin II was not affected. Collectively, our novel results revealed that Cdk1-dependent KIF4A phosphorylation at S1186 is a trigger for chromosomal organization during early mitosis.

## Introduction

In eukaryotes, DNA is highly compacted into chromosomes during cell division. Indeed, chromosome condensation is essential for the faithful segregation of chromosomes from one generation to the next. This process is achieved through the concerted action of various condensation factors, including chromosome-binding proteins [1–3], post-translational modifications (PTMs) [4] and certain cations [5, 6]. A key step in the organization of the rod-shaped chromatid is the formation of chromatin loop arrays during early mitosis [7]. These chromatin loop arrays are arranged along the axial structure located in the center of the chromatid, named “chromosome scaffold”. The chromosome scaffold structure was first observed as a network of non-histone proteins in histone-depleted metaphase chromosomes by electron microscopy [8]. Since then, several proteins in the scaffold have been identified, including complexes of condensin I and II [9, 10], topoisomerase IIα [11], and kinesin family member 4A (KIF4A) [12]. Topoisomerase IIα, the most abundant protein in the chromosome scaffold, has been suggested to contribute to axial shortening during chromosome formation [13]. Condensin I and II are involved in the formation of chromatin loops to varying degrees and cause lateral compaction and axial shortening of chromatids, respectively [7, 14].

KIF4A, known as a chromokinesin, is a type of motor protein kinesin that binds to both microtubules and chromosomes [15]. Several studies have revealed that the localization of KIF4A in the chromosome scaffold is similar to condensin and topoisomerase IIα [12, 13, 16, 17]. However, how the motor protein is involved in chromosome condensation remains unclear. KIF4A depletion causes chromosomes to be wider and shorter, suggesting the probable action of KIF4A in the lateral compaction of chromosomes [13]. In addition, KIF4A interacts with condensin I, and the accumulation of KIF4A and condensin I in the chromosome scaffold is interdependent [17–19]. This suggests that the lateral compaction of the chromatid is achieved concertedly by both condensin I and KIF4A. Condensin I accumulates in the scaffold through the motor activity of KIF4A [17], but how the dynamics of KIF4A are controlled during chromosome condensation remains poorly understood.

Previous studies have indicated that phosphorylation of scaffold proteins regulates the chromosome organization. In the initial stage of chromosome condensation, Cdk1 phosphorylates the condensin II subunit CAP-D3 at T1415, which triggers mitotic chromosome assembly by recruiting another kinase, Plk1, to the chromosome scaffold [20]. Msp1 regulates the localization of condensin II through the phosphorylation of CAP-H2 at S492 [21]. After nuclear envelope breakdown (NEB), aurora B kinase phosphorylates the condensin I subunit CAP-H at S70 [22] for its loading to the chromosome. The phosphorylation of CAP-G at T308/332 by Cdk1 also serves to regulate the DNA-binding ability of condensin I [23]. For KIF4A, its localization to the chromosome and its interaction with condensin I are both controlled by several mitotic kinases [18, 19, 24]; however, the functional meaning of KIF4A phosphorylation remains unclear.

In this study, we demonstrated that the phosphorylation of KIF4A at S1186 is required for its chromatin-binding ability during mitosis. In addition, this phosphorylation depends on Cdk1 activity. Lack of KIF4A phosphorylation at S1186 showed a phenotype similar to that of chromosomes observed in KIF4A-knockdown cells. In this phenotype, condensin I is diffused from the chromosome and the shape of the chromosome changes to be both wider and shorter. Collectively, our novel results suggest that the phosphorylation of KIF4A at S1186 by Cdk1 triggers lateral chromatin compaction by loading condensin I onto the chromosome scaffold.

## Materials and methods

### Cell culture

HeLa Tet-On 3G, the human cervical cancer cell line, was purchased from Clontech, and maintained in Dulbecco’s modified Eagle’s medium (DMEM) supplemented with 10% fetal bovine serum (FBS) and 800 μg/mL G418 in a humidified atmosphere of 5% CO_2_ at 37°C. For the kinase inhibition experiments, HeLa cells were cultured in the medium containing 0.1 μg/mL nocodazole for 14 h to arrest the cell cycle at mitosis, and further treated with 10 μM MG132 for 1 h. The mitotic cells were then treated with 10 μM RO-3306 (RO), 4 μM ZM447439 (ZM), or 100 nM BI2536 (BI) for 2 h. Cells expressing EGFP-KIF4A WT or EGFP-KIF4A S1186A were cultured in the medium containing 1 μg/mL doxycycline and 2 μg/mL puromycin.

### Antibodies

The following primary antibodies were used for immunostaining (IF) and western blotting (WB): rabbit polyclonal anti-KIF4A (IF-1:500, WB-1:500; Thermo), rabbit polyclonal anti-phosphorylated KIF4A S1186 (WB-1:500), rabbit polyclonal anti-CAP-H1 (IF-1:100, WB-1:300), rabbit polyclonal anti-GFP (WB-1:1000; Thermo), rabbit polyclonal anti-CAP-H2 (WB-1:500), rat polyclonal anti-CAP-H2 (IF-1:500; Cosmo bio), rabbit polyclonal anti-CAP-D2 (WB-1:500), rabbit polyclonal anti-CAP-D3 (WB-1:500), rabbit polyclonal anti-histone H3S10 phosphorylation (WB-1:2000; Upstate), chicken polyclonal anti-GFP (IF-1:1000; Abcam), rabbit polyclonal anti-histone H3 (WB-1:1000; Millipore), and mouse monoclonal anti-α-tubulin (WB-1:1000; Calbiochem). Anti-CAP-H1, anti-CAP-H2, anti-CAP-D2, anti-CAP-D3 and anti-phosphorylated KIF4A S1186 antibodies were produced in rabbits by immunization against CAP-H1 [GTEDLSDVLVRQGD], CAP-H2 [KRFQTYAAPSMAQP], CAP-D2 [TTPILRASARRHRS], CAP-D3 [SRRSLRKTPLKTA], and KIF4A [KKTPPAP(pS)PFDLPE] peptides, respectively (Eurofins).

### Plasmid construction

The EGFP-KIF4A expression vector was constructed by cloning the amplified KIF4A cDNA fragment into the pEGFP-C1 plasmid. KIF4A cDNA that was originally cloned inside the pF1K vector was purchased from the Kazusa DNA Research Institute. The KIF4A cDNA fragment was first amplified using primers that introduced *Bgl*II and *Sal*I restriction enzyme sites into both ends of the KIF4A sequences, followed by purification and digestion with *Bgl*II (NEB) and *Sal*I (NEB). The pEGFP-C1 plasmid was also digested with the same restriction enzymes. The digested KIF4A cDNA fragment was then cloned into the digested pEGFP-C1 plasmid by using a DNA ligation kit (Takara). The pEGFP-C1-KIF4A plasmid was then digested with *Nhe*I and *Sma*I, and inserted into the pTRE 3G plasmid (Clontech) digested with *Eco*RV using a DNA Blunting Kit (Takara).

To obtain KIF4A phosphorylation mutants, serine (S) or threonine (T) residues at four KIF4A phosphorylation sites (T799, S1001, T1181, and S1186) were substituted with alanine (A) or aspartic acid (D), which contains GCT or GAC DNA sequences, using a PrimeSTAR mutagenesis basal kit (Takara). All EGFP-KIF4A WT and mutant plasmid DNA sequences were confirmed.

### Transfection

HeLa Tet-On 3G cells were transfected with pTRE3G-EGFP-KIF4A WT or mutant plasmids using Lipofectamine 3000 (Invitrogen). Linear puromycin marker DNA (Takara) was also transfected to select EGFP-KIF4A-expressing cells.

To knockdown endogenous KIF4A and CAP-D2, HeLa cells were transfected with 150 nM KIF4A siRNA (5′-CAGGTCCAGACTACTACTC-3′) and 150 nM CAP-D2 siRNA (5′-CCAUAUGCUCAGUGCUACATT-3′) using Lipofectamine RNAiMAX (Invitrogen). After incubation for 48 h, the cells were collected and used for analysis.

### Immunostaining and fluorescence microscopy

In HeLa cells expressing EGFP-KIF4A WT or mutants, endogenous KIF4A was depleted by RNAi treatment. To prepare chromosome spreads, cells were treated with 0.1 μg/mL colcemid for 14 h, and mitotic cells suspended in PBS were spun onto coverslips coated with poly-L-lysine (Matsunami) by cytocentrifugation (Shandon Cytospin 4, Thermo) at 1,300 rpm for 10 min. The chromosome spreads or cells cultured on the poly-L-lysine-coated coverslip were fixed with 2% para-formaldehyde (PFA) in PBS for 15 min and permeabilized with 0.2% Triton X-100 in PBS for 5 min at room temperature (RT). Samples were blocked with 1% bovine serum albumin in PBS for 30 min at RT. Samples were then incubated with primary antibodies at RT for 1 h, followed by secondary antibodies labeled by Alexa Fluor 488 or 594 at RT for 1 h. DNA was counterstained with Hoechst 33342. Samples were mounted onto glass slides using Vectashield mounting medium (Vector Laboratories) or p-phenylenediamine.

Images were taken with a DeltaVision microscope (AppliedPrecision) equipped with a 1.42 NA PlanApo N 60× oil immersion objective (Olympus) and a cooled CCD camera (cool SNAP HQ2, Photometrics). The z-stack distance was 0.2 μm; raw 3D images were deconvoluted using constrained iterative deconvolution and converted to 2D images with maximum projection using softWoRx (AppliedPrecision). Confocal images were obtained with an A1R+ system (Nikon) with GaAsP detectors under the small pinhole setting (0.4AU) and applied deconvolution (Nis-Elements C-ER, Nikon) to enhance resolution. Laser lines at 405 nm for Hoechst 33342, 488 nm for GFP, and 561 nm for Alexa 594 were used for excitation. An objective lens with NA of 1.49 (SR ApoTIRF 100 ×, oil, Nikon) was used. The brightness and contrast of the images were adjusted using ImageJ software.

### Live cell imaging

Cells were cultured in poly-L-lysine-coated 35-mm glass bottom dishes (Matsunami). Before observation, the DNA was counterstained with Hoechst 33342 for 15 min, followed by replacing the medium with phenol red-free DMEM containing 10% FBS, 2 mM L-glutamine, and 20 mM HEPES. Time-lapse observation was performed using a Delta Vision microscope (Applied Precision) equipped with a CO_2_ chamber set at 37°C. A 1.42 NA PlanApo N 60× oil immersion objective (Olympus) was used to observe cells at 3-min time intervals.

### Cell and chromatin extraction and WB

Whole cell extracts (WCE) were obtained by sonicating cells in 1× Laemmli sample buffer after washing with PBS. For chromatin extraction, cells were washed with PBS and the cell pellets were re-suspended in freshly prepared CSK buffer (10 mM PIPES, pH 6.8, 10% glycerol, 3 mM MgCl_2_, 100 mM NaCl, 1 mM DTT, 0.25 mM PMSF, and 0.3% Triton X-100) supplemented with Complete protease inhibitor (Roche) and PhosSTOP phosphatase inhibitor mixture (Roche), followed by incubation for 30 min on ice. Insoluble chromatin pellets were collected by centrifuging at 16,100 *g* for 10 min at 4°C. The supernatant was mixed with the same volume of 2× Laemmli sample buffer and collected as chromatin-unbinding proteins (CUB). Insoluble chromatin pellets were washed again with CSK1 buffer. The chromatin pellets were re-suspended in 1× Laemmli sample buffer and collected as chromatin-binding proteins (CB) after sonication. All samples were heated to 95°C for 5 min and subjected to SDS-PAGE using 5%–10% or 5%–20% gradient polyacrylamide gels (Nacalai). Proteins were transferred to PVDF membranes (Millipore) using a PoweredBlot Ace transfer system (ATTO). The membranes were then blocked by Bullet Blocking One (Nacalai). Primary and secondary antibodies were diluted in Signal Enhancer HIKARI for WB and ELISA (Nacalai) and reacted with proteins at RT for 1 h. After each antibody reaction, the membrane was washed with TBST (20 mM Tris, pH 7.5, 150 mM NaCl, 0.1% [v/v] Tween 20). Immunoreactive bands of proteins were then detected using a 1% NBT/BCIP stock solution (Roche) in alkaline phosphatase buffer (100 mM Tris, pH 9.5, 100 mM NaCl, 5 mM MgCl_2_). The membrane image after band detection was captured using a WSE-5200 Printgraph 2M (ATTO). The brightness and contrast of the images were adjusted, and the band intensity was quantified using ImageJ software.

### Immunoprecipitation

For immunoprecipitation, endogenous KIF4A was depleted from HeLa cells expressing EGFP-KIF4A WT or S1186A by RNAi treatment. The cells were cultured in the medium containing 0.1 μg/mL nocodazole for 14 h, and mitotic cells were collected by shake-off. The cells were washed with PBS and cell pellets were re-suspended in lysis buffer (20 mM Tris-HCl, pH 7.5, 150 mM NaCl, 5 mM MgCl_2_, 20 mM β-glycerophosphate, 1 mM DTT, 5% glycerol, 0.1% NP-40) containing 0.1 μM okadaic acid, protease inhibitors (cOmplete, Mini, EDTA-free, Roche), and 2 U/μL OmniCleave endonuclease (Ar Brown), and incubated on ice for 20 min. Insoluble chromatin pellets were collected by centrifuging at 15,000 rpm for 10 min, with the supernatant collected as the mitotic extract (input). Mitotic extracts were rotated with anti-GFP antibody (rabbit polyclonal, Thermo) or anti-SMC2 antibody (rabbit polyclonal, Abcam) for 1 h at RT. As a negative control, the antibody was reacted with a mitotic extract prepared from HeLa cells without expressing GFP-KIF4A. The antibody-binding complex was recovered using Dynabeads (Thermo) and dissolved in 1× Laemmli sample buffer. The immunoprecipitates were then analyzed by WB.

### Image quantification and statistical analysis

All images were deconvolved using SoftWoRx (Applied Precision) before further analysis using ImageJ software. After subtraction of cytoplasmic DNA and target protein signals, signal intensity of the target protein in the stained DNA region was obtained. The protein fluorescence intensity was normalized to the DNA fluorescence intensity as per following calculation: [protein intensity/DNA intensity]. Statistical analysis was carried out by performing Student’s t-test using Microsoft Excel software.

## Results

### Phosphorylation of KIF4A at S1186 is required for chromosome localization

**Fig 1:**
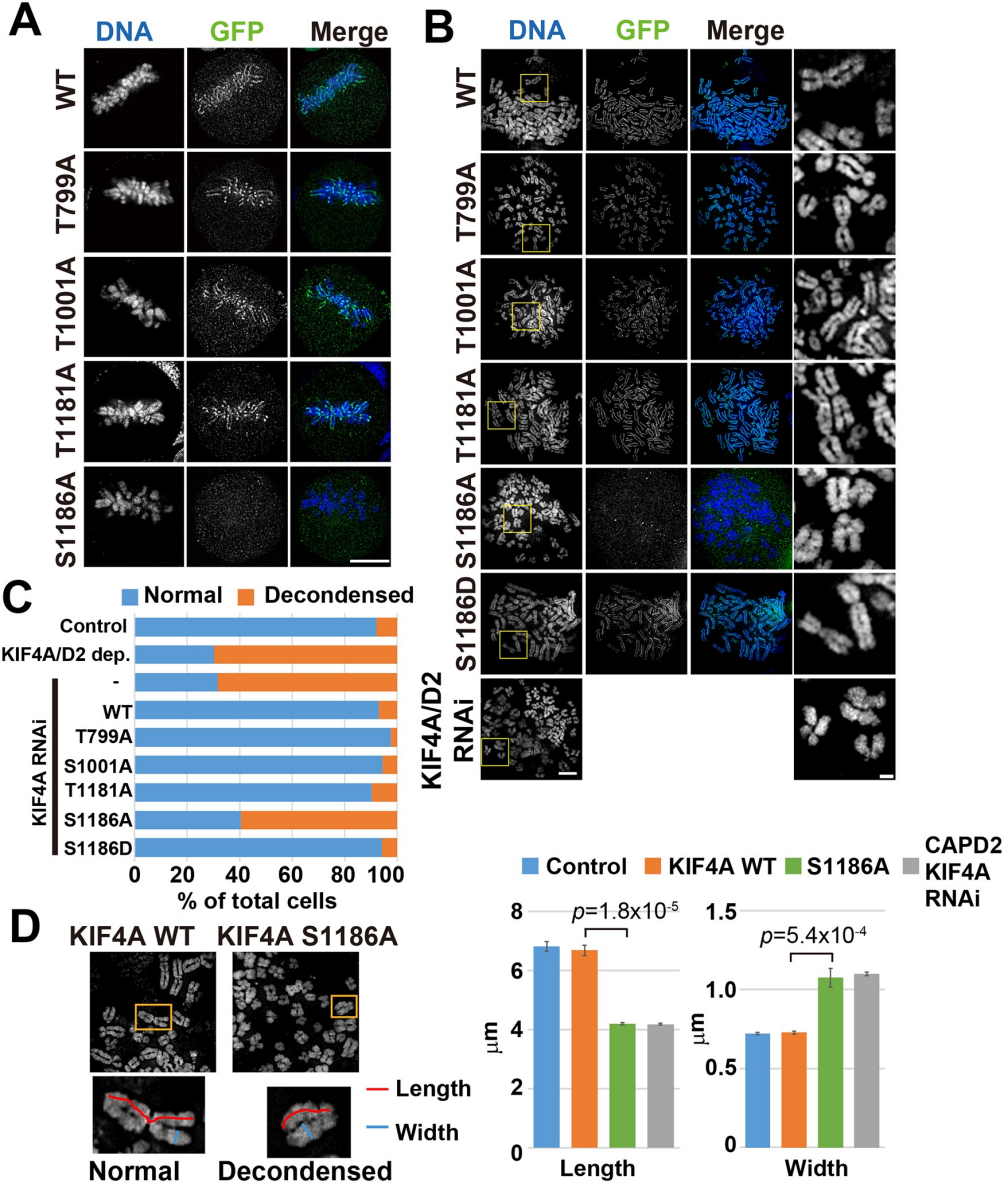
Phosphorylation of KIF4A S1186 is required for chromosome localization and chromosome morphology. (A) Localization of EGFP-KIF4A WT or non-phosphorylatablemutants in metaphase cells. Only the KIF4A S1186 mutant did not localize to the mitotic chromosomes. Bar, 5 μm. (B) Localization of EGFP-KIF4A WT and mutants on spread chromosomes. Localization of KIF4A along the chromosome scaffold disappeared in EGFP-KIF4A S1186-expressing cells. Endogenous KIF4A was depleted in all EGFP-KIF4A-expressing cells. As a control for chromosome decondensation, spread chromosomes prepared from KIF4A and CAP-D2, a condensin I subunit, double depleted cells are indicated in the bottom panels. Images of stained DNA in yellow boxes are enlarged in the rightmost panels. Bars, 5 μm for whole images, 1 μm for enlarged images. (C) Percentage of cells showing normal and decondensed chromosome morphology, as shown in (D), in control (non-transfected), KIF4A/CAPD2 dep. (KIF4A and CAPD2 RNAi) (non-transfected), EGFP-KIF4A WT-, T799A-, S1001A-, T1181A-, S1186A-, and S1186D-expressing cells. More than 30 cells were counted for the classification in each condition, and the percentage reported is an average of three independent experiments. (D) Comparison of the chromosome size between EGFP-KIF4A WT- and S1186A-expressing cells. Entangled chromosomes are enlarged at the bottom of each image. The length (red) and width (blue) of the chromatid were measured. As a control for chromosome decondensation, chromosome size in both KIF4A and CAP-D2- depleted cells was also examined. The six longest chromosomes in a single cell were manually measured using ImageJ; 20 cells were analyzed in each condition. The graph shows the average length and width from three independent experiments.

In this study, we analyzed the function of KIF4A phosphorylation during mitosis. Four phosphorylation sites of KIF4A, namely, T799, S1001, T1181, and S1186, were targeted. The phosphorylation of KIF4A at T799 during mitosis has been previously reported to be important for regulating the mitotic spindle [25]; however, its function during chromosome condensation is not known. The other three phosphorylation sites were identified as phosphorylated peptides that were found to be enriched in the condensed chromatin in our previous study. To determine the function of these phosphorylation sites, we constructed non-phosphorylatable mutants of KIF4A in which the amino acid residues serine and threonine were substituted with alanine. EGFP-fused KIF4A WT and the non-phosphorylatable mutants T799A, T100A, T1181A, and S1186A were expressed in HeLa cells, and their localizations at metaphase were observed under a fluorescent microscope (Fig 1A). In these cells, endogenous KIF4A was depleted by RNAi (S1A Fig). Although three KIF4A mutants, T799A, T1001A, and T1181A, showed accumulation in the chromosome scaffold similar to KIF4A WT, KIF4A S1186A did not localize to the chromosomes.

To examine the effects of KIF4A phosphorylation on chromosome morphology, spread chromosomes were prepared from EGFP-KIF4A WT- or non-phosphorylatable mutant-expressing cells. The chromosome morphology of EGFP-KIF4A T799A-, T1001A-, and T1181A-expressing cells was similar to that of KIF4A WT-expressing cells; however, chromosomes in EGFP-KIF4A S1186A-expressing cells showed expanded chromosome morphology, shorter chromosome length and wider width, which was also observed in both KIF4A and CAP-D2-depleted cells (Fig 1B and 1C). The phosphomimetic mutant of KIF4A S1186D could localize to chromosome similar to KIF4A WT, and no defects in chromosome morphology were observed (Fig 1B). These results indicate that KIF4A phosphorylation at S1186A is required for its localization in chromosomes and normal chromosome condensation. Other phosphorylation sites did not show defects in their localization or chromosome morphology; thus, we chose to focus on the phosphorylation of KIF4A at S1186.

### KIF4A phosphorylation at S1186 depends on Cdk1 activity during mitosis

**Fig 2:**
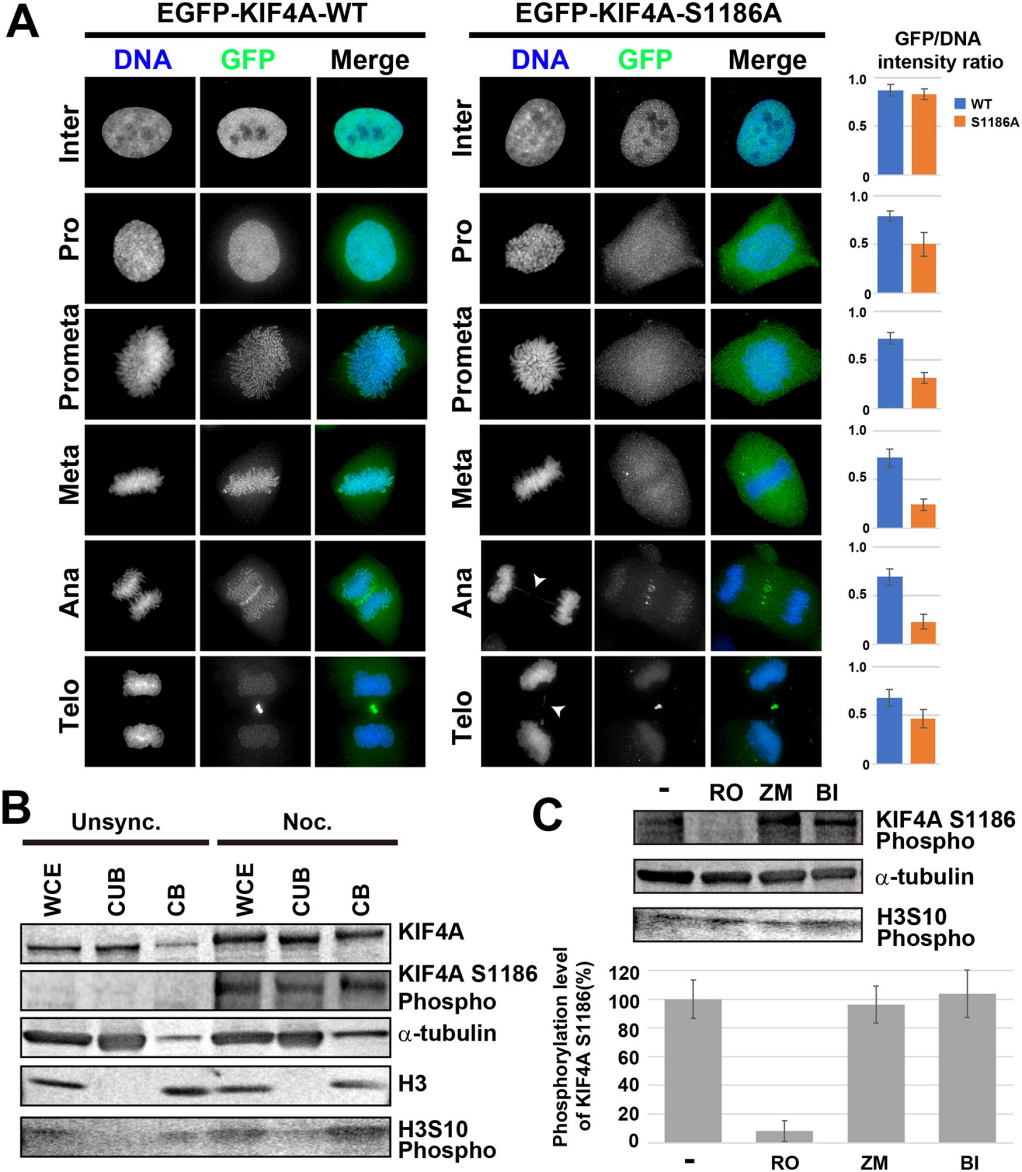
Cdk1-dependent phosphorylation of KIF4A at S1186 regulates the chromosomal localization of KIF4A during mitosis. (A) Localization of EGFP-KIF4A WT or S1186A was analyzed in fixed cells immunostained with anti-GFP (green) antibody. DNA was counterstained with Hoechst 33342 (blue). Arrowheads indicate chromosome bridge formation. Bar, 5 μm. EGFP-KIF4A WT localized to the chromosome, whereas the localization of EGFP-KIF4A S1186A to the chromosomes was severely decreased after NEB. Bar graphs show GFP/DNA intensity ratios in each mitotic stage (n = 50). (B) Unsynchronous and nocodazole-treated HeLa cells were fractionated into the chromatin-unbound fraction (CUB) and chromatin-binding fraction (CB), respectively, and whole cell extracts (WCE) were also analyzed. The phosphorylation of KIF4A at S1186 increased in mitotic cells, particularly in the chromatin-binding fraction. Alpha-tubulin and histone H3 were also detected as cytoplasmic protein and chromatin protein controls, respectively. Phosphorylation of histone H3 at S10 was detected to confirm the increase in mitotic cells. (C) Mitotic cells were treated with 10 μM RO-3306 (RO), 4 μM ZM447439 (ZM), or 100 nM BI2536 (BI) for 2 h. Cells were then corrected and whole cell extracts were analyzed by WB. Alpha-tubulin was detected as a loading control. KIF4A phosphorylation at S1186 was reduced by RO treatment, indicating that KIF4A S1186 was phosphorylated by Cdk1.H3S10 phosphorylation was detected as mitotic marker, but the intensity was decreased by ZM treatment, as it is phosphorylated by aurora kinase B. The band intensity of phosphorylated KIF4A S1186 was quantified (n = 3).

To estimate the function of KIF4A phosphorylation at S1186, the localization of EGFP-KIF4A S1186A was compared with that of EGFP-KIF4A WT throughout the cell cycle. In the interphase, both EGFP- KIF4A WT and S1186A were localized to the nucleus (Fig 2A). After nuclear envelope breakdown (NEB), some fractions of EGFP-KIF4A WT localized to the chromosome and accumulated in the chromosome scaffold, whereas EGFP-KIF4A S1186A was diffused in the cytoplasm (Fig 2A). The localization in the midbody at anaphase and telophase was not affected by defects in phosphorylation (Fig 2A). Similar localization patterns of EGFP-KIF4A WT and S1186A were also observed during live cell imaging (S2 Fig), which revealed that the loss of KIF4A phosphorylation at S1186 affects early mitotic progression. The duration of early mitosis (from NEB to anaphase onset) was increased from 38.5 ± 9.2 min (WT, n = 20) to 63.2 ± 13.7 min (S1186A, n = 20). Furthermore, in EGFP-KIF4A S1186-expressing cells, chromosome bridge formation was observed more frequently (34.4% in anaphase and telophase cells, n = 100; arrowheads in Fig 2A) than that in EGFP-KIF4A WT-expressing cells (10.9% in anaphase and telophase cells, n = 100), suggesting that KIF4A phosphorylation at S1186 is required for regular chromosome segregation.

To determine the timing of KIF4A phosphorylation at S1186, a rabbit polyclonal anti-phosphorylated KIF4A S1186 was produced. This antibody detected EGFP-KIF4A WT, T799A, S1001A, T1181A, but not EGFP-KIF4A S1186A (S1B and S1C Fig). KIF4A knockdown by RNAi also decreased the band intensity detected using this antibody in WB analysis (S1B Fig). These results indicated that the antibody specifically recognized KIF4A phosphorylation at S1186. HeLa cells were arrested at mitosis by nocodazole treatments, and the phosphorylation level of KIF4A at S1186 was compared with that in unsynchronized cells. In mitotic cells, phosphorylation levels of KIF4A S1186 were drastically increased, particularly in the chromatin-binding fraction, whereas almost no bands were detected in unsynchronized cells (Fig 2B). This finding indicates that the phosphorylation of KIF4A at S1186 is mitotic-specific.

Next, we determined the kinase that phosphorylates KIF4A at S1186. There are three major kinases known to be involved in protein phosphorylation during mitosis: Cdk1, aurora kinase B, and Plk1. Mitotic-arrested cells were treated with the kinase inhibitors RO-3306 (Cdk1 inhibitor), ZM447439 (aurora kinase B inhibitor), or BI2536 (Plk1 inhibitor). Then, the phosphorylation level of KIF4A at S1186 was then detected by WB. Phosphorylation levels were drastically decreased in Cdk1-inhibited cells, whereas phosphorylation levels were largely unchanged in the other kinase-inhibited cells (Fig 2C). These findings indicated that KIF4A phosphorylation at S1186 depends on Cdk1 activity.

### KIF4A phosphorylation at S1186 is required for the accumulation of condensin I in the chromosome scaffold

**Fig 3:**
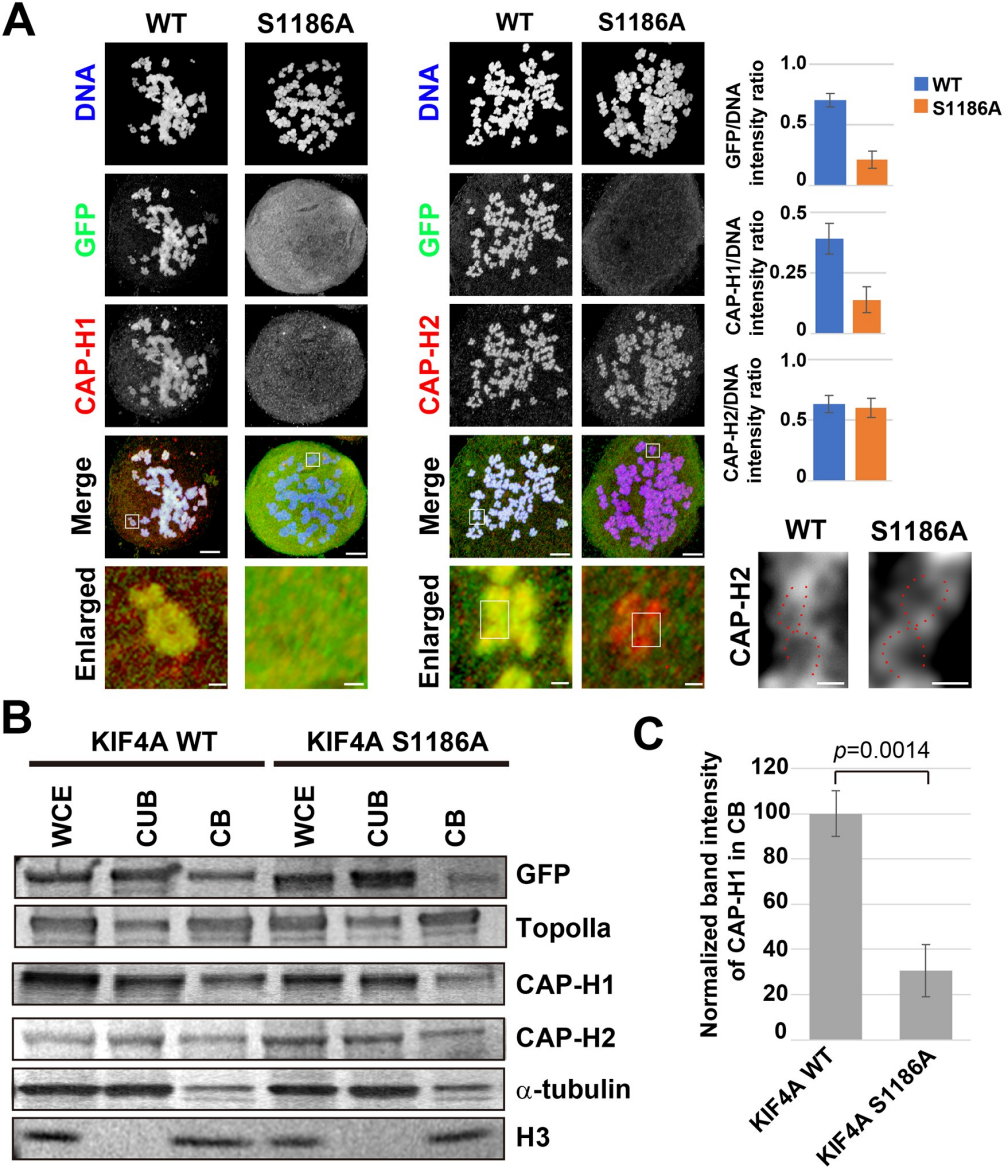
Effects of the loss of KIF4A phosphorylation at S1186 on the chromosome scaffold. (A) Localization of EGFP-KIF4A and condensin subunits. Chromosome spreads were prepared from mitotic cells expressing EGFP-KIF4A WT or S1186A. Endogenous KIF4A was depleted by RNAi. EGFP-KIF4A (green), and a condensin I subunit CAP-H1 (red) and a condensin II subunit CAP-H2 (red) were detected by immunostaining. DNA was counterstained with Hoechst 33342 (blue). Fluorescent signals were observed by confocal microscopy. Bottom panels show enlarged images of chromosomes in white boxes without DNA signals. The localization of CAP-H2 in the white box in enlarged images was further magnified and the image contrast was changed to clarify the scaffold structure. DCS was observed in both KIF4A WT and KIF4A S1186A-expressing cells (red broken line). Bar graphs show the signal intensity ration of GFP, CAP-H1 and CAPH-2 on chromosomes vs. DNA (n = 20). Bars, 5 μm in whole cell images, 300 nm in enlarged images, and 150 nm in DCS images. (B) The amounts of chromosome scaffold proteins in EGFP-KIF4A WT- or S1186A- expressing cells. Mitotic cells were fractionated into the chromatin-unbound fraction (CUB) and the chromatin-binding fraction (CB). Whole cell extracts (WCE) were also analyzed. The amounts of the chromosome scaffold proteins GFP-KIF4A, topoisomerase IIα (TopoIIα), CAP-H1, and CAP-H2 were estimated by WB analysis. Alpha-tubulin and histone H3 were also detected as cytoplasmic protein and chromatin protein controls, respectively. This panel is organized from three membranes and all blots were shown in S3 Fig. (C) The band intensity of CAP-H1 in the CB was normalized and compared between the EGFP-KIF4A WT- and S1186A-expressing cells (n = 3).

As shown above (Fig 1D), the chromosome morphology in EGFP-KIF4A S1186A-expressing cells was expanded and was similar to that reported in condensin I-depleted cells [19]. Because previous studies have shown the interdependency of KIF4A and condensin I on chromosome localization [17–19], the localization of condensin I was examined in spread chromosomes prepared from EGFP-KIF4A S1186A-expressing cells without endogenous KIF4A. To observe the detailed chromosome scaffold structure, as shown in our previous report [16], a confocal microscope with near SIM resolution (~150 nm) was employed. In EGFP-KIF4A WT-expressing cells, both a condensin I subunit CAP-H1 and a condensin II subunit CAP-H2 were detected in the chromosome scaffold with KIF4A (Fig 3A). However, in EGFP-KIF4A S1186A-expressing cells, both KIF4A and CAP-H1 were diffused into the cytoplasm and no accumulation in the chromosomes was observed (Fig 3A). In contrast, the localization of CAP-H2 was not affected by the defect in KIF4A phosphorylation, and the double-stranded chromosome scaffold (DCS) could be observed (Fig 3A, enlarged). These results indicate that KIF4A phosphorylation at S1186 is required for the accumulation of condensin I in the chromosome scaffold, but is not likely important for condensin II to form DCS.

The amounts of the chromosome scaffold proteins were examined by WB analysis. The chromatin-binding ability of KIF4A was decreased in the S1186A mutant compared with the KIF4A WT (Fig 3B, GFP). Similar to our microscope observation results, the chromatin binding of CAP-H1 was significantly decreased in KIF4A S1186A-expressing cells, but the binding of CAPH2 and another chromosome scaffold protein, topoisomerase IIα, was not affected (Fig 3B and 3C).

### KIF4A phosphorylation at S1186 regulates the interaction with the condensin I complex

**Fig 4:**
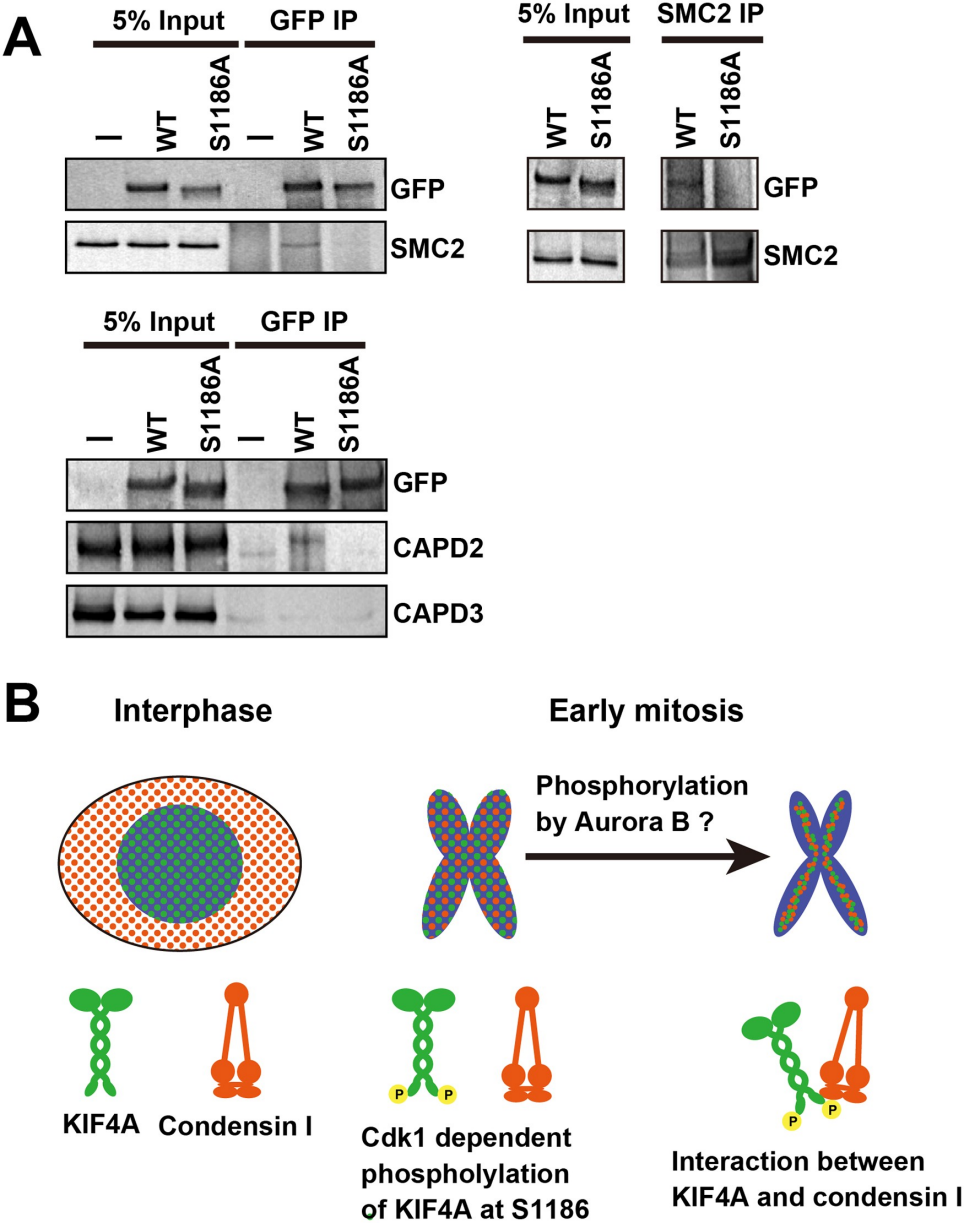
The interaction between KIF4A and condensin I. (A) HeLa cells expressing EGFP-KIF4A WT or S1186 were arrested at mitosis and the KIF4A-condensin I complex was recovered by immunoprecipitation against GFP and SMC2. Mitotic extracts prepared from no EGFP expressing cells (-) were also analyzed as a negative control. To detect KIF4A and condensin, the anti-GFP, -CAPD-2, -CAP-D3, and -SMC2 antibodies were used for WB analysis. (B) A model of the chromosome organization by KIF4A phosphorylation. In interphase, KIF4A and condensin I localize in the nucleus and cytoplasm, respectively. In early mitosis, KIF4A is phosphorylated at S1186 by Cdk1, and associates with the chromosome. After NEB, condensin I is also distributed to the chromosome. KIF4A and condensin I then would form a complex in the chromosome by their further phosphorylation by aurora B. KIF4A assists in the accumulation of condensin I in the chromosome scaffold using its motor activity, and lateral chromatid compaction is achieved.

Because KIF4A interacts with the condensin I complex, and the lack of KIF4A phosphorylation at S1186 resulted in the delocalization of these proteins from chromosomes, their interaction may be regulated by KIF4A phosphorylation. To examine this issue, mitotic cell extracts were prepared from EGFP-KIF4A WT- or S1186A-expressing cells, and immunoprecipitation against GFP or SMC2 was performed. In EGFP-KIF4A WT-expressing cells, EGFP-KIF4A co-precipitated with SMC2 and a condensin I subunit, CAP-D2, but the amount was decreased in S1186A-expressing cells (Fig 4A). There was no interaction between EGFP-KIF4A and a condensin II subunit, CAP-D3. It indicates that KIF4A interacts with only condensin I as reported previously [17–19], and this interaction is regulated by KIF4A phosphorylation at S1186.

## Discussion

KIF4A and condensin are major chromosome structural proteins identified in our proteomics study of human metaphase chromosomes [2, 3]. Both proteins form a chromosome scaffold structure that serves to assemble the long chromatin fiber into the compact rod-shaped chromatid [13, 16]. Chromosome condensation during mitosis is believed to be achieved by chromatin loop formation along the chromosome scaffold [7], and KIF4A may assist in the accumulation of condensin in the scaffold using its motor activity [17]. In this study, we reveal that the phosphorylation of KIF4A at S1186 is required for its interaction with condensin I and their accumulation in the chromosome scaffold.

Protein phosphorylation is known to regulate protein function in many cellular activities, including chromosome condensation [20, 26]. For KIF4A, several mitotic phosphorylation sites have been previously identified [18, 24, 25]. KIF4A (also known as a chromokinesin) has multiple functions during mitosis, including chromosome condensation, mitotic spindle formation, and cytokinesis [27]. The combination of phosphorylation sites in KIF4A enables the tight regulation of its functions. In this study, four phosphorylation sites of KIF4A, namely, T799, S1001, T1181, and S1186, were functionally analyzed to determine their role in chromosome condensation. Although only KIF4A S1186 was found to be associated with chromosome condensation, other phosphorylation sites may be related to other mitotic KIF4A functions.

The loss of KIF4A phosphorylation at S1186 caused defects in the accumulation of both KIF4A and condensin I in the chromosome scaffold (Fig 3A), and the chromosome structure became expanded (Fig 1). These results are similar to previous reports of the effects of KIF4A depletion showing a wider and shorter chromosome phenotype and causing a reduction in condensin I in chromosomes [13, 19]. Consistent with these reports, this chromosome phenotype has also been observed when condensin I is depleted from cells [28]. Defects in chromosome segregation observed in EGFP-KIF4A S1186A-expressing cells is also related to the loss of condensin I from the chromosome [28, 29]. Because KIF4A phosphorylation at S1186 is required for its interaction with condensin I, the KIF4A phosphorylation likely ensures their stable binding to chromosomes and correct chromosome condensation (Fig 4B). However, a recently identified phosphorylation site of KIF4A, T1161, is also required for the localization of KIF4A and condensin I to chromosomes; however, the fusion of the non-phosphorylatable mutant of KIF4A with histone H1 restored this interaction and the localizations [18]. Therefore, there is a possibility that KIF4A phosphorylation at S1186 is required only for its targeting to the chromosome after NEB, and KIF4A interacts with condensin I in chromosomes under the regulation by other factors, consistent with our previous observation [19].

Cdk1 forms a complex with cyclin B and promotes mitosis by protein phosphorylation. KIF4A has at least two phosphorylation sites for Cdk1, namely, T1161 [18] and S1186. If either T1161 or S1186 is not phosphorylated, KIF4A cannot localize to the chromosome after NEB. This indicates that phosphorylation at these sites is essential for the function of KIF4A after NEB, and this function is strictly regulated by multiple phosphorylation sites. Defects in phosphorylation sites causes a delay in early mitotic progression [12, 18]. The main function of KIF4A on chromosomes is likely to carry condensin I to the chromosome scaffold using its motor activity. In this regard, the function of KIF4A may not be directly related to chromosome condensation, because chromosome defects in cells lacking KIF4A phosphorylation by Cdk1 are similar to those of condensin I-depleted cells (Fig 1 and [18]). As condensin I distributes in the cytoplasm during interphase, KIF4A phosphorylated by Cdk1 serves to guide condensin I to the chromosome scaffold after NEB. This likely occurs through an interaction at the KIF4A C-terminal tail domain and the CAP-G subunit of condensin I [17].

KIF4A localizes not only to the chromosome scaffold, but also to the spindle midzone during late mitosis (Fig 2A), and it functions in midzone organization via its interaction with an anti-parallel microtubule-bundling factor, PRC1 [30]. KIF4A translocates PRC1 to the midbody, and the translocation is negatively regulated by the phosphorylation of PRC1 by Cdk1 [31]. The phosphorylation of KIF4A at T799/S801 by aurora B kinase and AMP-activated protein kinase is also known to promote the interaction between KIF4A and PRC1 [32, 33]. A faint GFP signal was detected on the mitotic spindle in EGFP-KIF4A S1186A-expressing cells, but not in EGFP-KIF4A WT-expressing cells (Fig 2A), implying that phosphorylation of KIF4A at S1186 controls its localization between the microtubule and the chromosome. Although we did not examine the function of KIF4A in the midbody formation, phosphorylation of KIF4A at S1186 by Cdk1 may prevent an untimely interaction between KIF4A and PRC1 by separating their localization to the chromosome and the spindle, respectively.

In addition to Cdk1, KIF4A is phosphorylated by aurora B and Plk1, although these phosphorylation sites have not been identified. The phosphorylation of KIF4 by aurora B is required for its interaction with condensin I [24], and this interaction is observed in the chromosome, but not in the cytoplasm [19]. Approximately 70% of KIF4A keeps its localization in the chromosome even after aurora B kinase inhibition [19], whereas its chromosome localization is almost completely lost in KIF4A non-phosphorylatable mutants phosphorylated by Cdk1 (Fig 2A). Thus, the phosphorylation by Cdk1 likely guides KIF4A to the chromosome, then phosphorylation by aurora B would established its interaction with condensin I to allow it to accumulate in the chromosome scaffold (Fig 4B). Plk1 phosphorylation is probably required for the dissociation of KIF4A from the chromosome [19]. Therefore, the sequential phosphorylation of KIF4A by Cdk1, aurora B, and Plk1 appears to be required for correct chromosome condensation and decondensation during mitosis.

## Supporting information

S1 Fig Confirmation of KIF4A knockdown and anti-KIF4A antibody specificity. (A) The expression of endogenous KIF4A was suppressed by siRNA transfection against 3’ UTR of KIF4A (lower band). However, the expressions of GFP-KIF4A WT and S1186A were not affected by siRNA transfection (upper band). (B) The mitotic cell extracts were subjected to WB analysis using anti-GFP (upper panel), anti-KIF4A S1186 phosphorylation (middle panel), and anti-α-tubulin antibodies. Using anti-KIF4A S1186A phosphorylation antibody (middle panel), three bands were detected. The middle band (indicated by *) was a non-specific band, because the band was appeared in the cell extracts prepared from KIFA knockdown cells. The upper band shows GFP-KIF4A S1186 phosphorylation levels, but it also includes background signal derived from non-specific proteins detected in untransfected HeLa cells. The signal intensity of GFP-KIF4A S1186A-expressing cells was similar to that of untransfected cells, indicating the KIF4A mutant was not phosphorylated at S1186. The lower band shows phosphorylation level of endogenous KIF4A S1186 and the intensity was decreased by KIF4A knockdown. (C) The specificity of anti-KIF4A S1186 phosphorylation antibody was confirmed using cell extracts prepared from GFP-KIF4A WT-expressing cells and non-phosphorylatable mutants. The band detected by anti-KIF4A S1186 phosphorylation antibody disappeared only in EGFP-KIF4A S1186A-expressing cells.
(TIF)

S2 Fig Dynamics of EGFP-KIF4A WT and S1186A during mitosis. (A) HeLa cells expressing EGFP-KIF4A WT or S1186A were observed from prophase to telophase of mitosis. EGFP-KIF4A WT showed localization on chromosomes throughout mitosis, and its accumulation at midbody was observed in late mitosis. In contrast, EGFP-KIF4A S1186A did not localized on chromosomes after nuclear envelop breakdown, and diffused into cytoplasm. The accumulation at midbody in late mitosis was observed similar to that observed for EGFP-KIF4A WT. (B) The GFP/DNA signal intensity ratios at each mitotic stage in living cells were obtained from EGFP-KIF4A WT and S1186A-expressing cells (n = 20).
(TIF)

S3 Fig Detection of chromosome scaffold proteins by WB. All blotting results used in Fig 3B are shown here.
(TIF)
