# Supplementary figures and images for: Cdk1-dependent phosphorylation of KIF4A at S1186 triggers lateral chromosome compaction during early mitosis
Source: PLoS One. 2018 Dec 21;13(12):e0209614. doi: 10.1371/journal.pone.0209614 (PMC6303012; doi:10.1371/journal.pone.0209614)

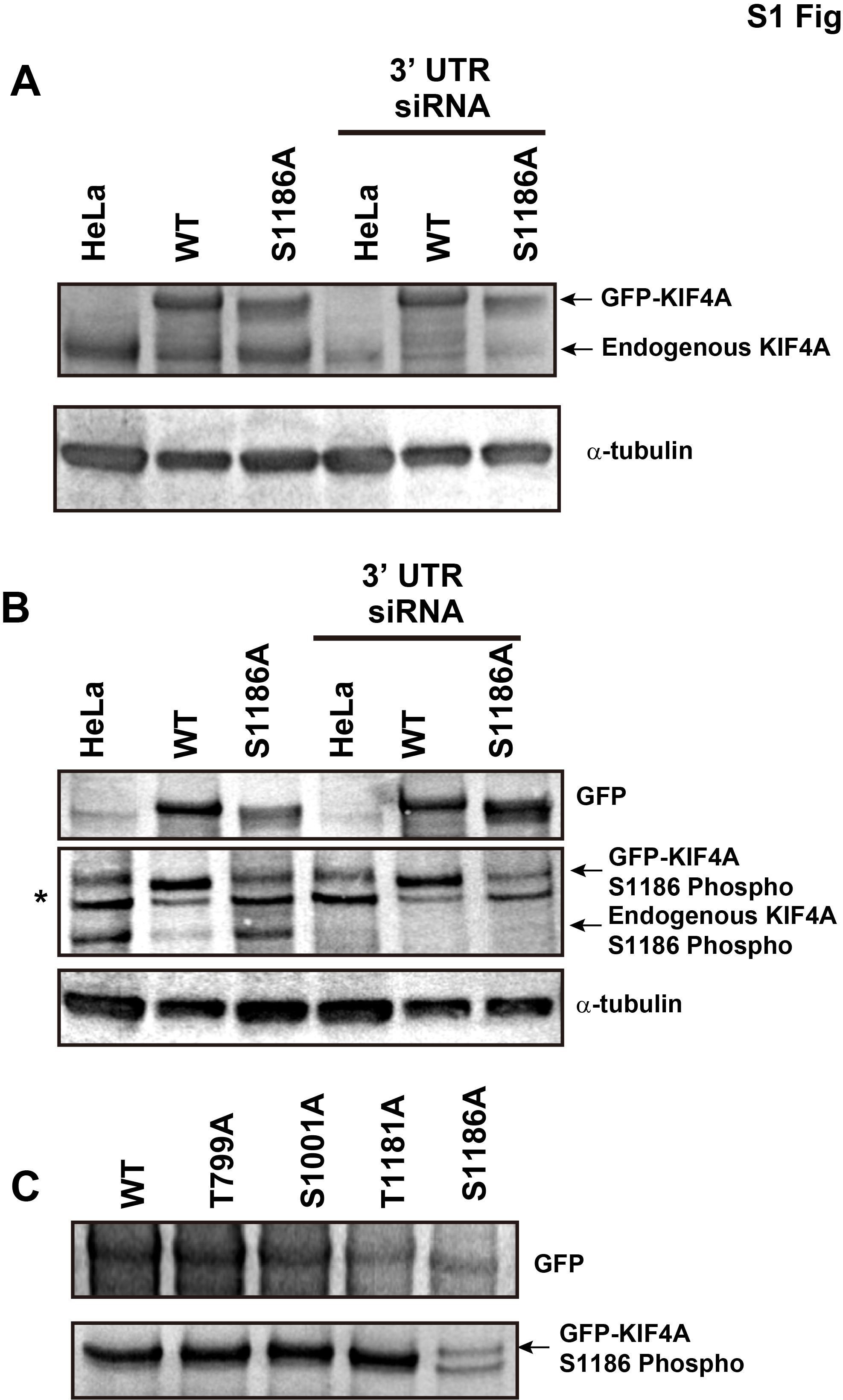

Supplement: S1 Fig — (A) The expression of endogenous KIF4A was suppressed by siRNA transfection against 3’ UTR of KIF4A (lower band). However, the expressions of GFP-KIF4A WT and S1186A were not affected by siRNA transfection (upper band). (B) The mitotic cell extracts were subjected to WB analysis using anti-GFP (upper panel), anti-KIF4A S1186 phosphorylation (middle panel), and anti-α-tubulin antibodies. Using anti-KIF4A S1186A phosphorylation antibody (middle panel), three bands were detected. The middle band (indicated by *) was a non-specific band, because the band was appeared in the cell extracts prepared from KIFA knockdown cells. The upper band shows GFP-KIF4A S1186 phosphorylation levels, but it also includes background signal derived from non-specific proteins detected in untransfected HeLa cells. The signal intensity of GFP-KIF4A S1186A-expressing cells was similar to that of untransfected cells, indicating the KIF4A mutant was not phosphorylated at S1186. The lower band shows phosphorylation level of endogenous KIF4A S1186 and the intensity was decreased by KIF4A knockdown. (C) The specificity of anti-KIF4A S1186 phosphorylation antibody was confirmed using cell extracts prepared from GFP-KIF4A WT-expressing cells and non-phosphorylatable mutants. The band detected by anti-KIF4A S1186 phosphorylation antibody disappeared only in EGFP-KIF4A S1186A-expressing cells. (TIF) [file pone.0209614.s001.tif]

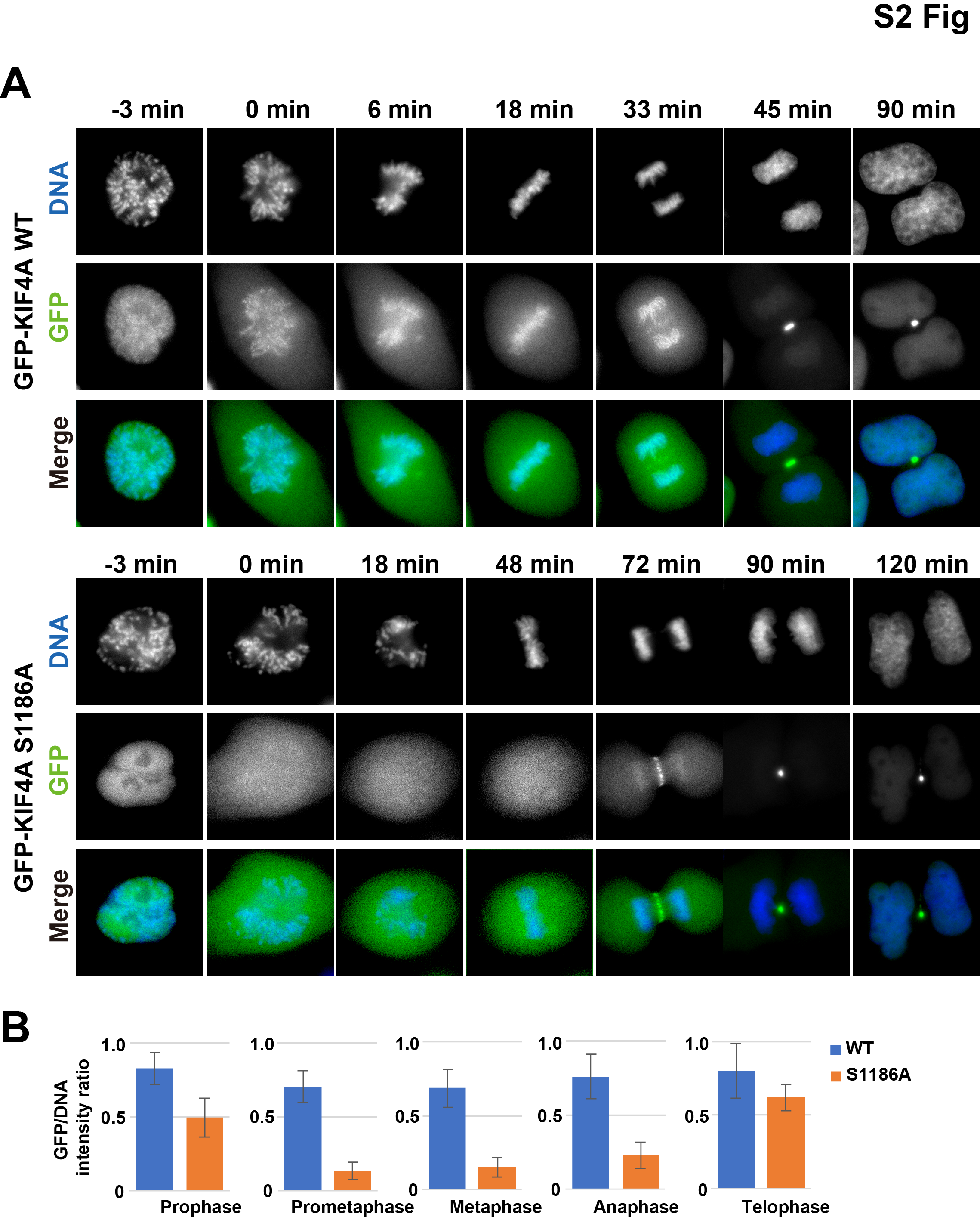

Supplement: S2 Fig — (A) HeLa cells expressing EGFP-KIF4A WT or S1186A were observed from prophase to telophase of mitosis. EGFP-KIF4A WT showed localization on chromosomes throughout mitosis, and its accumulation at midbody was observed in late mitosis. In contrast, EGFP-KIF4A S1186A did not localized on chromosomes after nuclear envelop breakdown, and diffused into cytoplasm. The accumulation at midbody in late mitosis was observed similar to that observed for EGFP-KIF4A WT. (B) The GFP/DNA signal intensity ratios at each mitotic stage in living cells were obtained from EGFP-KIF4A WT and S1186A-expressing cells (n = 20). (TIF) [file pone.0209614.s002.tif]

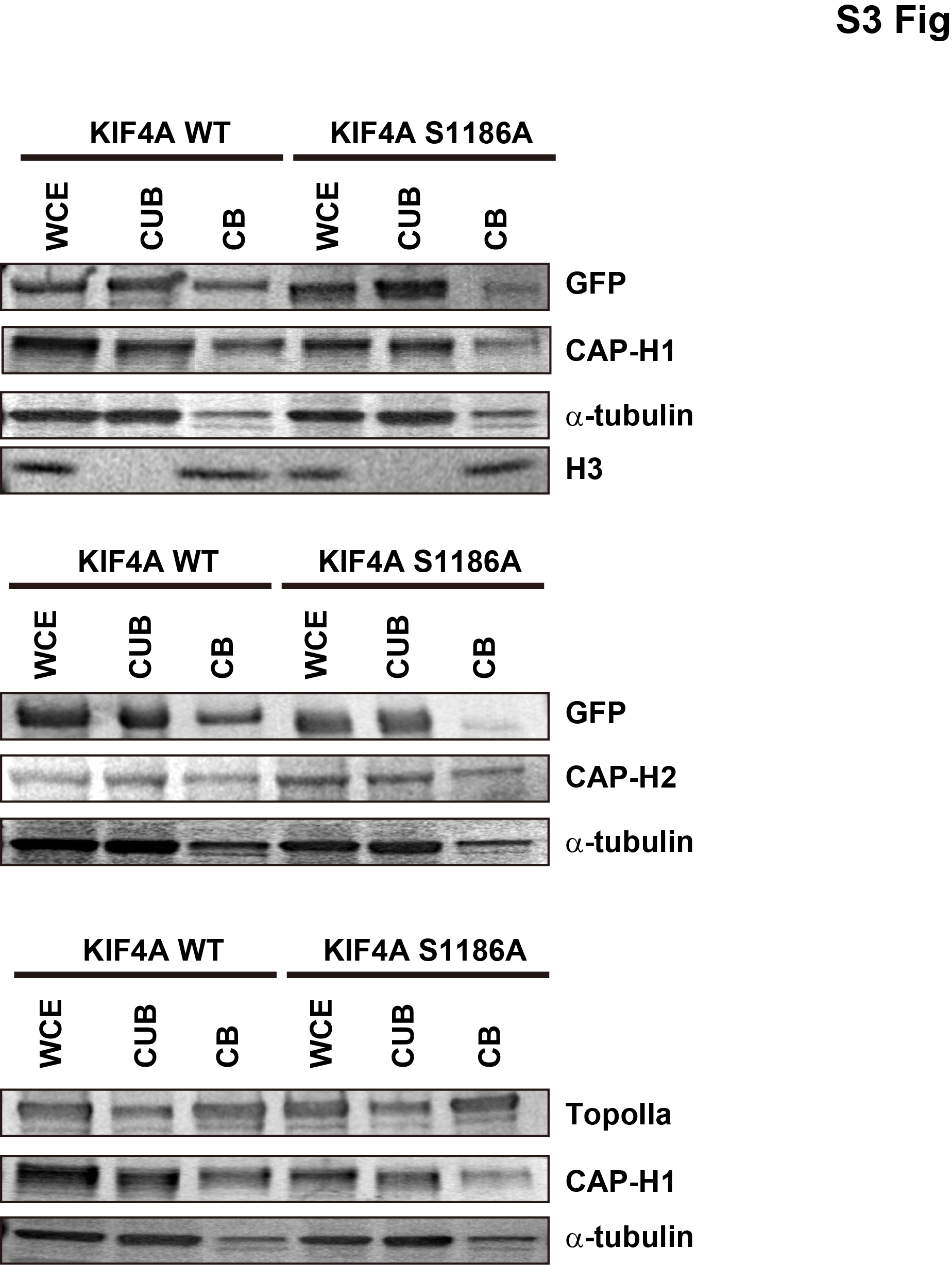

Supplement: S3 Fig — All blotting results used in Fig 3B are shown here. (TIF) [file pone.0209614.s003.tif]
